# Effects of Noise Exposure and Ageing on Anxiety and Social Behaviour in Zebrafish

**DOI:** 10.3390/biology12091165

**Published:** 2023-08-24

**Authors:** Raquel O. Vasconcelos, Flora Gordillo-Martinez, Andreia Ramos, Ieng Hou Lau

**Affiliations:** 1 Institute of Science and Environment, University of Saint Joseph, Macao, China; 2 MARE–Marine and Environmental Sciences Centre/ARNET—Aquatic Research Network, Faculdade de Ciências, Universidade de Lisboa, 1749-016 Lisbon, Portugal; 3 EPCV–Department of Life Sciences, Lusófona University, 1749-024 Lisbon, Portugal

**Keywords:** noise pollution, acoustic stress, senescence, novel tank diving, social preference

## Abstract

**Simple Summary:**

As animals age, exposure to additional environmental stressors such as noise pollution might be more detrimental at the physiological and behavioural levels, but such an effect is not yet clear. In this study, we tested the effects of noise exposure for 24 h on young adults and old zebrafish. Based on different behavioural tests, we found that both noise and ageing caused anxiety responses when the animals were introduced to a novel environment as well as a tendency for social proximity. Such anxiety responses decreased when the animals were tested in groups. Since the old zebrafish already showed anxiety-like behaviour with a preference for bottom dwelling, noise treatment induced the opposite effect in these individuals, increasing their vertical exploration. This work is a first attempt to investigate the effects of noise and ageing on zebrafish, a reference model in hearing and ecotoxicology research. Overall, we suggest that old individuals may have distinct physiological and behavioural mechanisms for dealing with noisy environments.

**Abstract:**

Noise pollution is creating a wide range of health problems related to physiological stress and anxiety that impact the social life of vertebrates, including humans. Ageing is known to be associated with changes in susceptibility to acoustic stimuli; however, the interaction between noise effects and senescence is not well understood. We tested the effects of 24 h continuous white noise (150 dB re 1 Pa) on both young adults and old zebrafish in terms of anxiety (novel tank diving test), social interactions (with mirror/conspecific attraction), and shoaling behaviour. Both noise and ageing induced higher anxiety responses in a novel environment. Since the old zebrafish showed longer bottom dwelling, acoustic treatment induced the opposite pattern with an initial increase in vertical exploration in the aged individuals. Both noise- and age-related anxiety responses were lowered when individuals were tested within a group. Regarding social interactions, both noise and ageing seemed to cause an increase in their proximity to a mirror. Although the results were not statistically significant, noise exposure seemed to further enhance conspecific attraction. Moreover, the interindividual distance within a shoal decreased with noise treatment in the aged individuals. This study is a first attempt to investigate the effects of both noise and ageing on zebrafish behaviour, suggesting the age-dependent physiological coping mechanisms associated with environmental stress.

## 1. Introduction

The physiological balance of an organism can be temporarily or permanently disrupted by environmental conditions, which occurs increasingly more often under the current scenario of global change [1,2,3]. The unprecedented levels of environmental noise due to growing anthropogenic activities are creating a serious hazard to the auditory systems of animals, including humans, but also a wide range of nonauditory effects, including cardiovascular problems [4,5], sleep disturbance [6], metabolic abnormalities [7], cognitive impairment [8], and poorer mental health and life quality [9,10]. Acoustic stress is also known to be associated with anxiety and/or depression in humans and can ultimately impact their social life [10,11].

Over the past decade, the literature has been rapidly expanding, demonstrating the effects of noise pollution on wildlife, including both terrestrial and aquatic organisms [12]. Increased noise levels can mask acoustic cues, distract attention, and induce stress responses, thus having the potential to affect behaviour [9]. While several behaviours, such as signalling, movement, foraging, vigilance, antipredator responses, and parental care, have been demonstrated to change under noise conditions in a wide range of taxa [13,14], less is known about social interactions. Nevertheless a few studies have demonstrated noise-induced effects on social communication, group dynamics, affiliation, and submission behaviour in various animal species, including fish [15,16,17,18].

How the body counteracts behavioural responses to acoustic disturbances is quite variable, depending on several factors, such as the type, intensity, and duration of noise exposure, but also on intrinsic individual differences, such as susceptibility and tolerance, that are highly dependent on age [12,19,20]. Ageing is a complex and multifactorial process that affects nearly every aspect of an organism’s physiology and behaviour [21], and it is known to be associated with changes in susceptibility/tolerance to acoustic stimuli in both human [22] and animal models [23]. Aged animals have higher inner-ear damage following acoustic trauma, more pronounced auditory shifts, and longer recovery times [20,24,25]. A few studies investigated the effects of noise and ageing on sound detection [26,27,28,29], but how both stress factors impact individual and social behaviour has never been investigated.

Animal models, such as zebrafish, have contributed to our understanding of physiological and behavioural responses to environmental stress [30,31], including the effects of noise pollution [32,33]. This model organism has also been key in investigating premature ageing, telomeropathies, and age-related neuropsychological disorders [34,35,36,37]. Our lab has established zebrafish as a model to investigate noise-induced hearing loss—NIHL [38,39,40]—and age-related hearing loss—ARHL (unpublished data). The present work investigated the effects of both ageing and noise on individual behaviour (anxiety in a novel environment) and social interactions (social preference and shoaling) in the zebrafish model. 

## 2. Materials and Methods

### 2.1. Test Animals and Husbandry

In this study, we used wild-type adult zebrafish (AB line) that were reared at the fish facility at the University of Saint Joseph, Macao SAR. These fish were descendants of animals initially obtained from the China Zebrafish Resource Centre (CZRC, Wuhan, China). Fish were housed in a stand-alone system (Model AAB-074-AA-A, Yakos65, New Taipei City, Taiwan) equipped with 10 L tanks containing filtered and aerated water (balanced pH of 7–8, conductivity of 400–550 μS) at 28 ± 1 °C. Animals were maintained under a 12:12 light/dark cycle and were fed twice daily with live Artemia and dry powdered food (Zeigler, Gardners, PA, USA). The housing protocol considered a standard of 20–30 fish per tank (2–3 fish/L in 10 L tanks) with a sex ratio of 2–3 males:1 female. For breeding purposes, we used breeding boxes placed inside the stock tanks, ensuring the offspring were from mixed progenitors.

A total of 72 zebrafish were used, belonging to two distinct age categories: young reproductive adults (4–7 months, 22–31 mm total length) and old (>24 months, 36–40 mm total length). The latter group included potential progenitors of the younger cohort. Individuals from each age category were assigned into different acoustic treatments (noise versus silent lab conditions/control), such as young control (YC), young noise (YN), old control (OC), and old noise (ON). A total of 18 individuals were used per experimental group for some behavioural tests (novel tank diving); however, for other assays (mirror/conspecific interaction test and shoaling), a lower number of individuals (*N* = 8–12) were considered for specific groups (YC and YN) due to adaptations of the experimental protocol.

All experimental procedures complied with the ethical guidelines regarding animal research and welfare enforced at the Institute of Science and Environment, University of Saint Joseph, and approved by the Division of Animal Control and Inspection of the Civic and Municipal Affairs Bureau of Macao (IACM). 

### 2.2. Noise Treatments

**Figure 1:**
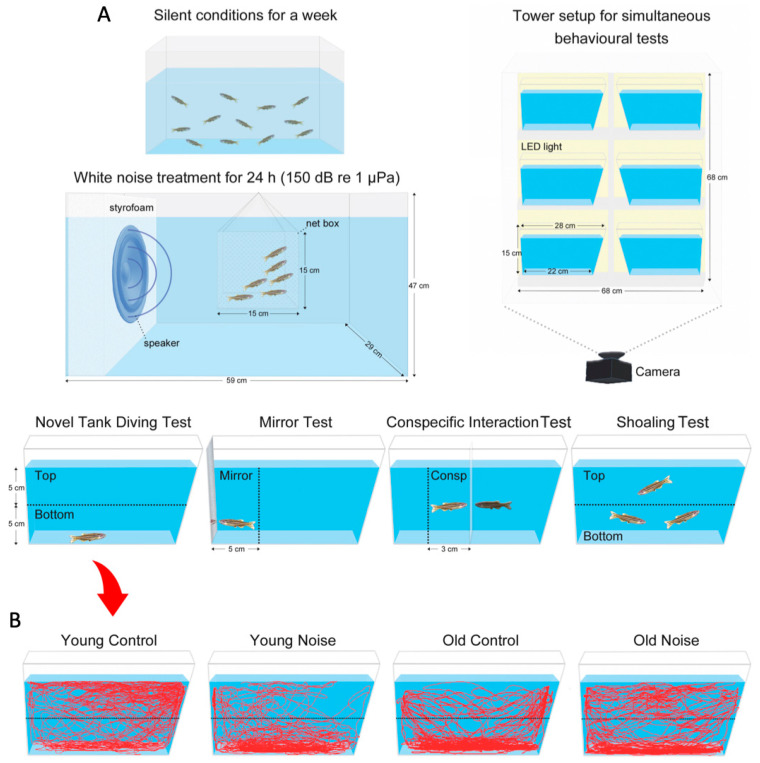
Experimental design showing the acoustic treatment followed by behavioural assays. (**A**) Young adult (4–7 months) or aged (>24 months) zebrafish were first maintained in silent laboratory conditions for 7 days, then transferred to treatment tanks for 24 h acclimation, and finally exposed to white noise (150 dB re 1 μPA) for 24 h. A tower setup was used to test 6 zebrafish simultaneously for the following tests: novel tank diving (NTD), mirror and/or conspecific interaction, and shoaling. (**B**) Diagrams showing representative examples of 9 different fish tracking lines (red) based on the period 1–2 min in the NTD test. Blue colour represents water medium in the experimental tanks.

The methodology used for noise exposure and control followed previously described procedures from our lab [38,40]. All subjects were initially transferred from the fish facility to 4 L tanks and placed in a quiet laboratory environment (sound pressure level, SPL, between 103 and 108 dB re 1 µPa) for at least 7 days. These tanks had no filtration system or pumps to avoid additional noise; however, water changes were performed every 2 days, and light and temperature were kept identical to stock conditions. This adaptation step was critical to reduce potential noise effects from the housing system [41]. After this period, groups of 6 zebrafish were transferred to glass acoustic treatment tanks (dimensions of 59 cm L × 29 cm W × 47 cm H, 70 L), where they remained inside a mesh box (dimensions of 15 × 15 × 15 cm, 1 mm mesh) suspended underwater at 2 cm distance from the speaker—see Figure 1.

The treatment tanks were equipped with an underwater speaker (UW30, Electro-Voice, Burnsville, MN, USA) placed between two vertical Styrofoam plates with a hole in the centre, which were positioned on one side of the tank. Treatment tanks were placed on top of Styrofoam boards and over two granite slabs spaced by rubber pads to reduce uncontrolled vibration. Sound was generated via underwater speakers that were connected to amplifiers (ST-50, Ai Shang Ke, Hangzhou, China) and finally to a laptop running Adobe Audition 3.0 for Windows (Adobe Systems Inc., San Jose, CA, USA). The same conditions were created inside the tank for the control treatment, with the amplifier turned on but without sound playback. 

Sound recordings and SPL measurements were carried out with a hydrophone (Brüel & Kjær type 8104, Naerum, Denmark; frequency range of 0.1 Hz–120 kHz; sensitivity of—205 dB re 1 V/μPa) connected to a hand-held sound level meter (Brüel & Kjær type 2270). Acoustic playbacks were calibrated to achieve the desired noise level (±8 dB) at the centre of the mesh box. 

### 2.3. Behavioural Tests

All behavioural tests were performed between 10:00 and 14:00. A tower setup was designed based on a previous study by Audira et al. [42], which allowed the simultaneous recording of six small tanks (2 L trapezoid-shaped tanks from the zebrafish housing system)—see Figure 1 for details. An LED light panel (60 × 60 cm, Opple, Eindhoven, The Netherlands) covered with an acrylic panel for light diffusion (overall 800-lux light intensity) was placed behind the tower setup to increase visibility for fish tracking. Testing tanks were filled with system water to achieve about 10 cm depth. Individual fish from each group were subject to either three or four behavioural tests, starting always with the novel tank diving (NTD) test. This was followed by either mirror or conspecific interaction test, or both assays in random sequence. The shoaling test was performed at last using the previously tested fish. Individual zebrafish were carefully moved with a net between testing tanks and were overall subject to a recording period of 60 min plus extra 3 to 5 min for relocating the animals between successive tests. The water from the experimental tanks was replaced between trials to avoid potential effects from chemical cues.

All experiments were recorded with a digital camera (Sony HDR-PJ675, Tokyo, Japan, 1080 p up to 50 fps), and videos were analysed with EthoVision XT 15 (Noldus Information Technology, Wageningen, The Netherlands). This software was used to generate an automatic tracking line for each individual and to analyse general swimming parameters, such as total distance (cm)—overall swimming distance; maximum acceleration (cm/s^2^); and freezing (%)—percentage of time immobile divided by the total recording time.

### 2.4. Novel Tank Diving (NTD) Test

To evaluate the impact of noise and ageing on swimming activity and anxiety-like behaviour, fish from each experimental group were tested with the NTD assay [43] immediately following the acoustic treatment or silent control conditions.

Individual fish were transported to the experimental tank, and the behaviour was recorded for 30 min. To measure the vertical exploratory activity, the tank was divided horizontally into two equally sized areas (top and bottom)—see Figure 1. Besides the general characterization of swimming activity, we measured the time spent in the bottom zone (%)—amount of time in the bottom zone per each consecutive 5 min throughout the session. The latency to the top zone was not considered as in previous studies [41] because the introduction of the different fish in the multiple tanks may have interfered with this parameter.

### 2.5. Mirror/Conspecific Interaction Test

The social preference was assessed using a mirror—mirror test [44,45]—and another equally sized conspecific [46], which are well-known assays used on zebrafish [47].

For the mirror test, a small mirror fitting the tank wall was placed on one side, and the zebrafish behaviour was recorded for 10 min. The observation space was virtually divided into two zones: mirror zone (<5 cm from mirror) and far zone (>5–17 cm)—see Figure 1.

In the conspecific interaction test, the tank was vertically divided with a transparent glass partition into two equally sized areas. A period of 5 min was given for acclimation to the partition, and then a similar-sized conspecific was placed on the opposite side. The focal fish behaviour was recorded for 5 min. Whenever individuals were less than 3 cm away from the partition, they would be considered within the conspecific zone.

For these assays, specific parameters included time in the mirror zone (%) and time in the conspecific zone (%).

### 2.6. Shoaling Test

The shoaling test was conducted to investigate the effect of age/noise on animals moving within a group [48]. In this experiment, three previously tested individuals were placed in a new tank, and their individual behaviour was recorded for 10 min. The shoaling behaviour was assessed through general swimming characterization and was based on the average time spent in the bottom zone (%) at the individual level and average interfish distance—distance between the body in centre of the different individuals within a shoal.

### 2.7. Data Analysis

For the NTD test across different time points, we used a repeated measures ANOVA. The experimental groups were considered a between-subject factor, while the time at bottom for the sequential time points were the repeated measures (within-subject factor). The remaining behavioural comparisons across the four experimental groups were based on one-way ANOVAs followed by planned contrasts to focus on specific pairwise comparisons between YC-YN, YC-OC, and OC-ON. Additional two-way ANOVAs were performed to quantify potential interactions between age and acoustic treatments.

All assumptions for parametric analyses were confirmed by inspecting normal probability plots and performing Levene’s test for homogeneity of variances. Freezing times (%) were square rooted to meet assumptions. All statistical tests were performed using SPSS v25 (IBM Corp., Armonk, NY, USA).

## 3. Results

### 3.1. Anxiety-Related Novel Tank Diving

**Figure 2:**
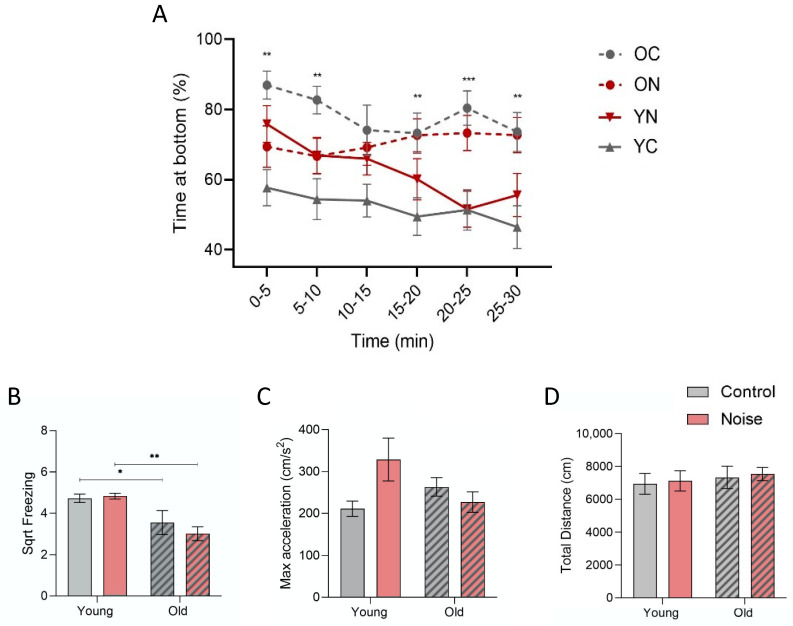
(**A**) Comparison of the time spent in the bottom zone during the novel tank diving (NTD) test by zebrafish adults – young and old (striped bars), after acoustic treatment (overall difference: F(3.68) = 6.709, *p* < 0.001). Significant differences were found at specific time points: 5, 10, 20, 25, and 30 min (F(3.68) = 4.351–8.209, *p* < 0.05). (**B**–**D**) Comparison of freezing (square rooted) (F(3.71) = 6.307, *p* < 0.001), maximum acceleration (F(3.71) = 2.698, *p* = 0.053), and total swimming distance (F(3.71) = 0.288, *p* > 0.05) between experimental groups based on the 30 min recording. Post hoc differences: * *p* < 0.05; ** *p* < 0.01; and *** *p* < 0.001.

We compared the effects of ageing and noise exposure on the anxiety-related behavioural patterns of adult zebrafish after being introduced to a novel environment. Our results showed overall differences between the experimental groups regarding the amount of time spent at the bottom of the tank over 30 min (F(3.68) = 6.709, *p* < 0.001)—see Figure 2A. These differences were significant at different time points, namely at 5, 10, 20, 25, and 30 min (F(3.68) = 4.351–8.209, *p* < 0.05). Out of all the groups, the noise-exposed individuals (YN) showed the highest change in the time spent at the bottom over the total recorded time (from 75.8% to 55.6%). Specifically, during the first 5 min, both ageing and noise caused an increase in the time spent at the bottom (F(3.68) = 5.718, *p* < 0.002) (YC vs. OC: *p* < 0.001) (YC vs. YN: *p* = 0.014). In the old zebrafish, however, the acoustic treatment induced the opposite pattern with less bottom dwelling (OC vs. ON: *p* = 0.018).

Moreover, the older individuals also displayed a lower freezing time (F(3.71) = 6.307, *p* < 0.001) and nearly significant changes in maximum acceleration (F(3.71) = 2.698, *p* = 0.053)—Figure 2B,C. All the groups revealed similar moving distances over the 30 min (F(3.71) = 0.288, *p* > 0.05)—Figure 2D. An additional analysis using a two-way ANOVA identified a significant interaction between age and acoustic treatment for the time spent at the bottom (during first 5 min, F(1.68) = 12.171, *p* = 0.001), contrary to freezing, max acceleration, and distance (F(1.68) = 0.001–0.791, *p* > 0.05).

### 3.2. Mirror/Conspecific Interaction

**Figure 3:**
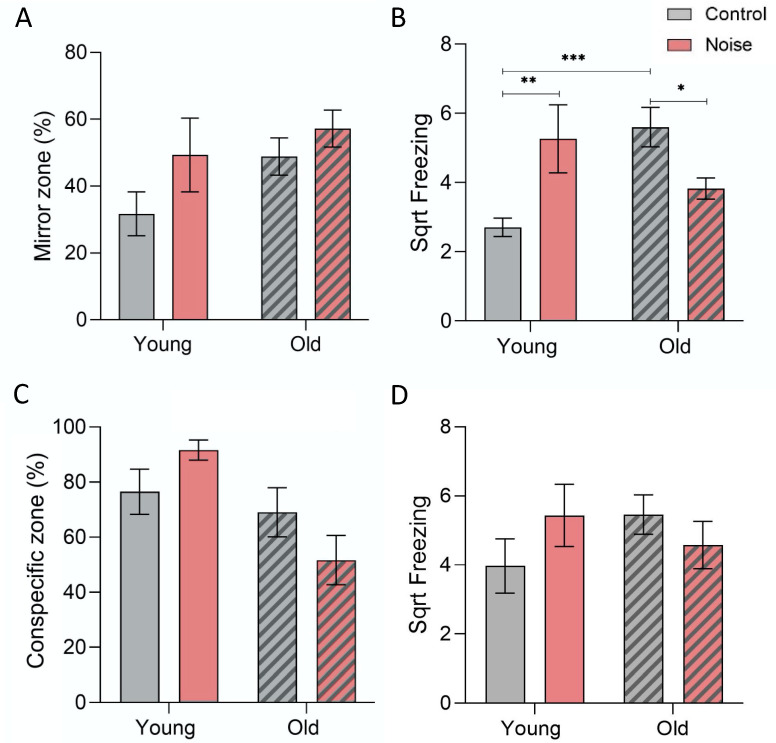
(**A**) Comparison of the time spent in the mirror zone by zebrafish adults – young and old (striped bars), after acoustic treatment (F(3.48) = 2.395, *p* = 0.081) and (**B**) freezing (F(3.52) = 5.191, *p* = 0.003) based on 10 min recording. (**C**) Comparison of the time spent in the conspecific zone by the same experimental groups (F(3.49) = 2.507, *p* = 0.071) and (**D**) respective freezing behaviour (F(3.49) = 0.818, *p* = 0.491) between the same experimental groups (based on 5 min). Post hoc differences: * *p* < 0.05; ** *p* < 0.01; and *** *p* < 0.001.

To assess the social preference, the adult zebrafish were tested regarding their interaction with a mirror. The comparison of the time spent closer to the mirror between the experimental groups was not significant (F(3.48) = 2.395, *p* = 0.081). Nevertheless, the older individuals showed a slight increase in their proximity to the mirror (YC vs. OC: from 31.7 to 48.9%), similar to the noise-exposed young (YC vs. YN: 31.7 to 49.3%) and old (OC vs. ON: 48.9 to 57.2%) groups—Figure 3A.

The interaction with the mirror also included freezing behaviour, which was significantly different among the groups (F(3.52) = 5.191, *p* = 0.003). Ageing alone induced an increase in the freezing time (*p* = 0.001) as well as the noise exposure (*p* = 0.010). In the aged individuals, however, the noise treatment caused the opposite trend with reduced freezing (*p* = 0.016)—Figure 3B. Hence, a significant interaction between age and acoustic treatment was identified for freezing (F(1.45) = 7.129, *p* = 0.011), contrary to the time spent closer to the mirror (F(1.45) = 0.429, *p* > 0.05).

When the mirror was replaced by another conspecific, we did not find overall statistical differences for the time spent closer to the other fish, but the results were close to significant (F(3.49) = 2.507, *p* = 0.071). Based on planned contrasts, noise alone induced a longer time spent close to the conspecific zone for the young group (YC vs. YN: *p* = 0.05), while ageing did not cause any changes (YC vs. OC: *p* > 0.05). Contrastingly, noise exposure promoted less time in the conspecific zone for the old zebrafish (*p* < 0.001). Freezing did not vary across the test groups (F(3.49) = 0.818, *p* > 0.05)—Figure 3C, D. For this behavioural assay, the ageing effects did not differ depending on the treatment (F(1.46) = 2.079–2.273, *p* > 0.05).

### 3.3. Shoaling Behaviour

**Figure 4:**
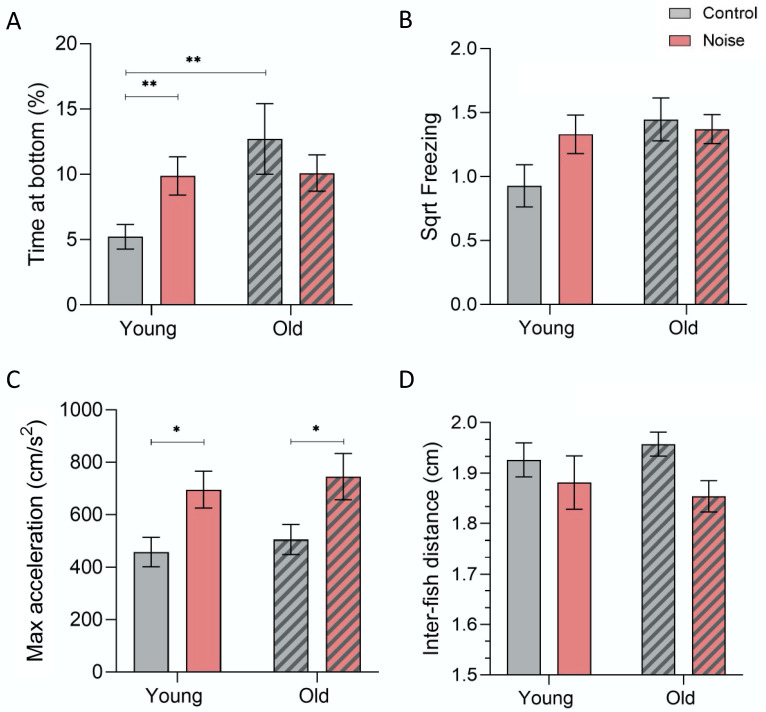
(**A**) Comparison of the time spent at the bottom of the tank (F(3.65) = 3.207, *p* = 0.029), (**B**) freezing (F(3.65) = 2.502, *p* = 0.068), (**C**) maximum acceleration (F(3.65) = 4.194, *p* = 0.009), and (**D**) interindividual distance (F(3.65) = 2.502, *p* = 0.068) between zebrafish adults – young and old (striped bars), after acoustic treatment (based on 10 min recording). Post hoc differences: * *p* < 0.05 and ** *p* < 0.01.

Locomotion during the shoaling test was significantly affected by noise exposure, namely in terms of swimming acceleration (F(3.65) = 4.194, *p* = 0.009). Noise caused an increase in the maximum acceleration in both the young adults and the old zebrafish (YC vs. YN: *p* = 0.028) (OC vs. ON: *p* = 0.014). Overall, freezing behaviour did not vary significantly (F(3.65) = 2.502, *p* = 0.068), but the aged individuals showed higher freezing within a shoal (*p* = 0.015)—Figure 4B,C.

**Figure 5:**
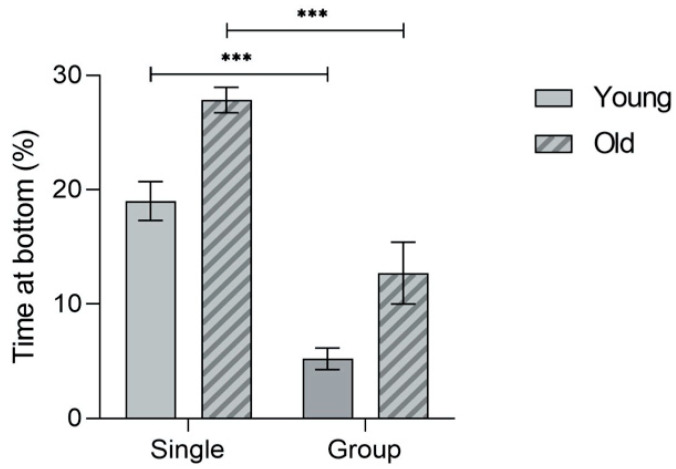
Comparison of the time spent at the bottom of the tank for both young adults and old zebrafish, when tested alone or within a group of 3 individuals (F(3.71) = 30.093, *p* < 0.001). Post hoc differences: *** *p* < 0.001. No interaction was identified between age and social context (F(1.68) = 0.148, *p* > 0.05).

Furthermore, both the aged and noise-exposed fish groups remained longer at the bottom of the tank (F(3.65) = 3.207, *p* = 0.029) (OC vs. YC: *p* = 0.016) (YN-YC: *p* = 0.015)—Figure 4A. This anxiety-like response was significantly lower when compared to the fish that were tested alone (F(3.71) = 30.093, *p* < 0.001) for both the young adults (*p* < 0.001) and the old fish (*p* < 0.001)—Figure 5.

Finally, the interindividual distances within a young group were not overall significantly different (F(3.65) = 2.502, *p* = 0.068), although aged individuals revealed shorter social distances after the acoustic treatment (*p* = 0.015)—Figure 4D.

Based on two-way ANOVAs, there were no significant interactions between age and acoustic treatment for the shoaling test (F(1.18–62) = 0–2.364, *p* > 0.05), although, for the time spent at the bottom, the results were nearly significant (F(1.62) = 3.886, *p* = 0.053).

## 4. Discussion

The present study represents a first attempt to investigate the effects of environmental noise and ageing on the zebrafish model. Our findings suggest that both noise exposure and ageing increased anxiety-like responses in a novel environment, which can be decreased by interaction within a group. The results obtained with the aged individuals that were previously exposed to noise conditions suggest distinct stress coping mechanisms and behavioural patterns associated with senescence.

### 4.1. Effects of Noise on Anxiety and Social Behaviour

We assessed the impact of noise on zebrafish behaviour using a well-established standardized assay to measure anxiety in this species, the novel tank diving (NTD) test. This assay exploits the innate tendency of zebrafish to dive to the bottom, seeking protection in a new environment, followed by a gradual increase in exploration as they start acclimating [43,49]. Novel environments are known to be anxiogenic for many animal species, including zebrafish, which reveal anxiety-related responses that can be modulated with anxiolytic drugs [42,43,50,51].

Our results showed that the noise-treated zebrafish spent more time in the bottom zone (>78%) during the first 5 min and then recovered vertical exploration until they reached levels similar to those of the control group (about 52% after 20 min). The control fish (i.e., the young adults under silent conditions) revealed comparatively less initial bottom dwelling (<60%) and thus fewer changes over time. Such results are in agreement with a previous study that investigated the effects of continuous and intermittent white noise at the same amplitude level (150 ± 10 dB re 1 μPa) on zebrafish [40]. According to this study, a similar difference (around 20%) between the noise and control groups was initially registered. This study, however, showed identical bottom times (or recoveries) after 3 min, and the swimming profile was only characterized within the first 5 min. Differences in the setups (tower for simultaneous recordings versus individual testing), the light conditions (a stronger background light in the tower setup), and manipulation (the tower setup implies moving tanks after the introduction of new fish) may explain the distinct recovery results.

Our findings suggest that the noise treatment caused anxiety-like behaviour in the zebrafish, similar to other studies that investigated the effects of anxiogenic treatments, such as caffeine (100 mg/L for 15 min [50]), ethanol (0.1% for 96 h [42]), or antibiotics such as oxytetracycline (10, 20, and 100 mg/L for 96 h [52]).

The noise-induced anxiety levels (based on the time at the bottom of the tank) decreased when the fish were tested within a group of three individuals. When exposed to a new environment, individual zebrafish are typically more stressed, showing higher cortisol levels and variable behaviour compared to fish within a group [53]. Like other teleost, zebrafish form shoals, and this strategy allows fast adaptation to a new environment, representing a functional unity that ensures social information exchange and protection from predators [53,54,55]. Hence, it is likely that noise-induced stress might be lowered by social interactions. Similarly, Audira et al. [42] also identified a lower anxiety level when zebrafish were in groups compared to their behaviour alone. These authors further showed that groups of three, four, and five were less anxious compared to fish in groups of two based on their NTD responses and increased interindividual distance.

Only a few studies have investigated how anthropogenic noise impacts social interactions in different fish species [15,16,18,56]. It was often assumed that fish shoals would respond to anthropogenic noise in a way that is analogous to antipredator behaviour; however, the findings are contradictory, showing that collective responses under acoustic stress are context- and species-specific and most likely depend on the type of noise disturbance and exposure regime [57,58]. For instance, pile-driving playback affected the shoal structure and dynamics in the seabass (*Dicentrarchus labrax*), inducing lower group cohesion and higher individual differences in spatial orientation and swimming speed [16]. Contrastingly, Currie et al. [57] tested the effect of tonal sounds (150 Hz of 1 s pulse) with different temporal patterns on the Eurasian minnow (*Phoxinus phoxinus*). These authors reported an increased group swimming speed, a decreased interindividual distance, and improved initial alignment in all the noise-exposed groups.

Our results suggest that noise exposure may increase the social preference (the proximity of an individual to a mirror or conspecific) and shorten the interindividual distances within a group, similar to Currie et al. [57]. Although our data showed a tendency, the results were not statistically significant, probably due to the sample size and/or methodological constraints with the multirecording setup. Moreover, the mirror test is often named the “mirror biting test” and is used to measure aggression [47]. In the present study, we did not quantify biting times, as we often found it difficult to be certain of an actual biting event. Even though our results point to a possible increase in aggressive displays (the proximity to the mirror) associated with noise exposure, further research is necessary to confirm this hypothesis.

Future work should investigate the noise-induced effects on zebrafish social behaviour considering a larger sample size, shoaling tests with more individuals, and varying noise regimes. Additionally, physiological stress should be confirmed through cortisol measurements.

### 4.2. Effects of Ageing on Anxiety and Social Behaviour

Ageing is a complex phenomenon that affects multiple aspects of an organism’s fitness and condition, including sensory, motor, and cognitive functions, and it is highly associated with the development of a wide range of neurodegenerative pathologies [21,59,60]. Zebrafish are commonly used in ageing research due to their short lifespan and genetic similarities to humans. Their gradual senescence process, evolutionarily conserved genome, and genetic tractability further enhance their suitability for such studies (reviewed by Van Houcke et al. [61]). Aged zebrafish are known to have declined basal locomotor activity [62], a reduced swimming ability [63], and an accentuated spinal curvature [64]. In the present study, we used individuals that were over 2 years old that displayed no apparent spinal curvature or other deformities that could adversely affect their swimming performance.

Our results showed that ageing caused a significant increase in anxiety-related behaviour in a novel environment. The old zebrafish revealed the longest time at the bottom (>85%) during the first 10 min of the NTD test and never recovered to the baseline levels of the young adults (remained at >70% after 30 min). Furthermore, the old individuals showed less freezing time in the NTD test, although the maximum acceleration and total moving distance were identical to those of the young adults. In social contexts, namely mirror interaction and shoaling, our results revealed increased freezing behaviour. Altogether, these results agree with Kacprzak et al. [65], who reported similar age-related anxiety responses (an increased overall time at the bottom of the tank) and a reduction in the swimming speed of zebrafish recorded for 24 h. Similarly, Evans et al. [66] reported a prolonged period of immobility and a longer time at the bottom of the tank for aged zebrafish during the initial 5 min of an NTD test in comparison to their younger adult counterparts. Such ageing effects were further investigated in terms of cognition by Yu et al. [67], who found increased stereotypic and reduced exploratory behaviour, changes in the cognitive responses to emotional experiences, a reduced generalization of adaptive associations, and altered temporal entrainment. Behavioural and cognitive differences that arise with age have been reported in several animal models, such as in mice [68] and birds [69]. Our findings provide further evidence that zebrafish can be used to investigate the ageing phenotype and associated behavioural disruption in both individual and social contexts.

### 4.3. Interaction between Ageing and Noise Exposure Effects

Ageing is known to be associated with changes in susceptibility or tolerance to acoustic stimuli [22,23]. Experiments with mammals exposed to acoustic trauma have shown that ageing is associated with stronger inner-ear damage, functional impairment, and delayed recovery [20,24,25]. However, previous studies have only evaluated the effects of both noise exposure and ageing on hearing [26,27,28,29]. We now investigated for the first time the effect of these stressors on individual and social behaviour in zebrafish.

Both age and noise alone induced higher anxiety responses in a novel environment. When the old zebrafish were exposed to the noise treatment, they showed the opposite pattern and spent less time at the bottom compared to the aged control. This pattern was verified in both the individual NTD and shoaling tests. The noise treatment also caused a reduction in freezing during mirror interaction and in the interindividual distance within a shoal in the aged individuals. Such behavioural changes most likely result from distinct age-dependent physiological coping mechanisms.

Our results are in accordance with Henríquez Martínez et al. [19], who found evidence that young and older zebrafish are differentially impacted by stress. The animals were exposed to a chronic (8-day) stress protocol that included the manipulation of the physical (temperature and water amount) and social parameters (crowding). According to these authors, however, while the young fish showed a significant change in anxiety-like behaviour (thigmotaxis), the older fish remained unaffected after such chronic stress. Nevertheless, our results are difficult to compare with this study since we focused on a single stressor that was applied for 24 h.

Limited knowledge is available on how age affects responses to environmental stress and on the underlying physiological control mechanisms. Distinct behavioural responses may arise from changes that occur in information processing and cognitive performance with senescence [68,70]. Age-related changes in coping strategies and perception thresholds have been reported and can ultimately influence how stress affects organisms [71,72]. Furthermore, prior experiences may also influence stimuli perception and interpretation, thus shaping behavioural outcomes [73].

## 5. Conclusions

Our findings suggest distinct stress coping mechanisms and behavioural responses associated with age and highlight that such an intrinsic factor is critical in experimental design and when interpreting the behavioural effects of noise exposure. Considering the rising levels of anthropogenic noise in aquatic ecosystems and the elevated chronic noise in housing facilities, it becomes critical to develop more comprehensive strategies to evaluate the impact of noise considering both species characteristics and age categories. The ongoing work investigates how noise and ageing impact auditory sensitivity and hormonal responses in zebrafish.

## Data Availability

The data presented in this study are available on request from the corresponding author.
